# Multimerin-2 is a ligand for group 14 family C-type lectins CLEC14A, CD93 and CD248 spanning the endothelial pericyte interface

**DOI:** 10.1038/onc.2017.214

**Published:** 2017-07-03

**Authors:** K A Khan, A J Naylor, A Khan, P J Noy, M Mambretti, P Lodhia, J Athwal, A Korzystka, C D Buckley, B E Willcox, F Mohammed, R Bicknell

**Affiliations:** 1 Molecular Angiogenesis Laboratory, Institutes of Biomedical Research and Cardiovascular Sciences, College of Medical and Dental Sciences, University of Birmingham, Birmingham, UK; 2 Rheumatology Research Group, Institute of Inflammation and Ageing, University of Birmingham, Birmingham, UK; 3 Cancer Immunology and Immunotherapy Centre, Institute of Immunology and Immunotherapy, University of Birmingham, Birmingham, UK

## Abstract

The C-type lectin domain containing group 14 family members CLEC14A and CD93 are proteins expressed by endothelium and are implicated in tumour angiogenesis. CD248 (alternatively known as endosialin or tumour endothelial marker-1) is also a member of this family and is expressed by tumour-associated fibroblasts and pericytes. Multimerin-2 (MMRN2) is a unique endothelial specific extracellular matrix protein that has been implicated in angiogenesis and tumour progression. We show that the group 14 C-type lectins CLEC14A, CD93 and CD248 directly bind to MMRN2 and only thrombomodulin of the family does not. Binding to MMRN2 is dependent on a predicted long-loop region in the C-type lectin domain and is abrogated by mutation within the domain. CLEC14A and CD93 bind to the same non-glycosylated coiled-coil region of MMRN2, but the binding of CD248 occurs on a distinct non-competing region. CLEC14A and CD248 can bind MMRN2 simultaneously and this occurs at the interface between endothelium and pericytes in human pancreatic cancer. A recombinant peptide of MMRN2 spanning the CLEC14A and CD93 binding region blocks CLEC14A extracellular domain binding to the endothelial cell surface as well as increasing adherence of human umbilical vein endothelial cells to the active peptide. This MMRN2 peptide is anti-angiogenic *in vitro* and reduces tumour growth in mouse models. These findings identify novel protein interactions involving CLEC14A, CD93 and CD248 with MMRN2 as targetable components of vessel formation.

## Introduction

Angiogenesis describes the formation of new blood vessels from existing ones and is an integral part of reproduction, embryonic development and wound healing. Although mostly dormant in healthy adults, it is a component of numerous pathologies including cancer, diabetic retinopathy and atherosclerosis.^1^ The ability to control angiogenesis can provide therapeutic value and understanding the underlying molecular events is critical in this pursuit.

The endothelial specific transmembrane glycoprotein CLEC14A has been identified as a tumour endothelial marker, due to its greater expression in tumour vasculature than vessels in healthy tissue.^2, 3, 4, 5^ The closely related CD93 is also overexpressed in tumour endothelium and studies confirm a role in tumour angiogenesis.^6, 7, 8^ CD248 (endosialin or TEM1) is not expressed by endothelium but is found on pericytes and tumour-associated fibroblasts of multiple tumour types.^9^ These three relatively understudied glycoproteins are part of the group 14 family of C-type lectin domain (CTLD) containing proteins.

There is limited information about the molecular pathways that CLEC14A and CD93 regulate, although functional data have demonstrated roles for both in endothelial migration and tube formation.^2, 5, 7^ CLEC14A was previously shown to bind an endothelial specific extracellular matrix (ECM) protein multimerin-2 (MMRN2), and antibodies disrupting this interaction retard angiogenesis and tumour growth, confirming its role in tumour development.^3, 10^ Furthermore, a meta-analysis of microarray data from over 1000 patient samples across three cancer types identified CLEC14A, CD93 and MMRN2 as core components of a proposed ‘tumour angiogenesis signature’.^6^ Likewise, CLEC14A and MMRN2 are both upregulated with tumour progression in spontaneous mouse tumours.^10^

CD248 has also been shown to have roles in angiogenesis, particularly in vessel regression during vascular patterning.^11^ CD248 has been described as a marker of pericytes associated with glioma vasculature,^12^ and is elevated in the stroma of many other tumours including colorectal, melanoma and glioblastoma.^13, 14, 15^ For these reasons, CD248 is actively being pursued as a cancer target with clinical trials underway.^16^

Here we investigate the interactions of the CTLD group 14 family with the CLEC14A ligand MMRN2 and show CLEC14A, CD93 and CD248 all engage MMRN2, whereas thrombomodulin of the family does not. Our findings propose previously unknown protein–protein interactions that occur in endothelium and the surrounding stroma, providing new targets in anti-angiogenic treatment.

## Results

### CTLD group 14 family members CLEC14A and CD93 directly bind MMRN2

**Figure 1:**
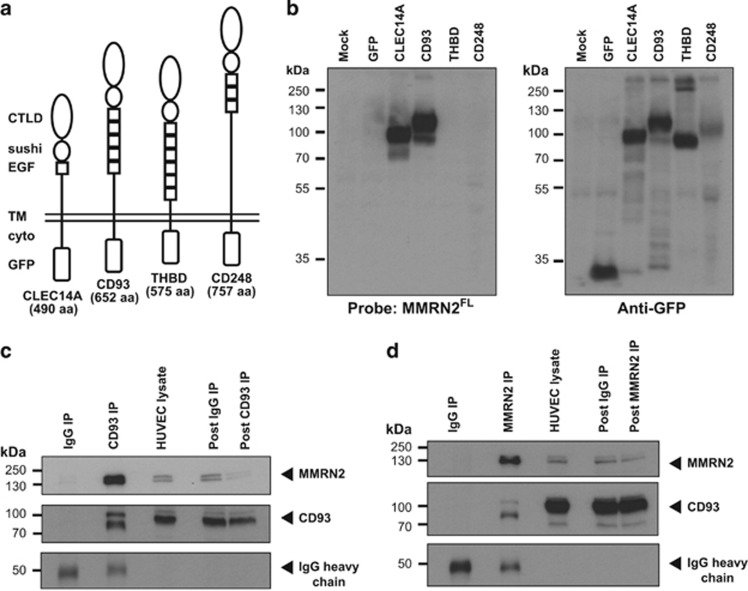
CD93 directly binds to MMRN2. (**a**) Diagrams of GFP-tagged CTLD group 14 family members showing domain architecture and relative size with number of amino acids (aa) without the GFP tag. C-type lectin domain (CTLD), sushi, epidermal growth factor (EGF) domain, transmembrane (TM), cytoplasmic tail (cyto) and green fluorescent protein (GFP) tag are displayed. (**b**) HEK293T were transfected with GFP-tagged group 14 family members, lysates were separated by SDS–PAGE under non-reducing conditions and far western blotted with MMRN2 full-length (MMRN2^FL^), showing MMRN2^FL^ binds CLEC14A and CD93 but not thrombomodulin or CD248, probing with anti-GFP confirmed expression of all proteins. (**c**) Immunoprecipitations of CD93 using monoclonal R139 antibody co-immunoprecipitates MMRN2 from HUVEC lysates. (**d**) Immunoprecipitations of MMRN2 using mouse polyclonal antibodies co-immunoprecipitates CD93 from HUVEC lysates. CD93 was detected using goat polyclonal antibodies in each immunoprecipitation experiment. IgG heavy chains included as loading control.

We previously identified MMRN2 as a CLEC14A-binding partner,^3^ to examine whether other CTLD group 14 members also bind MMRN2, we used far western blotting using a MMRN2 protein probe to test for direct protein–protein interactions. The CTLD group 14 members CLEC14A, CD93, thrombomodulin and CD248 were constructed with C-terminal green fluorescent protein (GFP) tags (Figure 1a), transfected into HEK293T cells and lysates were separated by SDS–PAGE under non-reducing conditions maintaining disulphide bonds. Transferred polyvinylidene fluoride membranes were probed using HEK293T lysates overexpressing full-length (FL) MMRN2 (MMRN2^FL^) with a polyhistidine (His) tag. MMRN2^FL^ bound to CLEC14A and CD93 detected by His tag antibodies (Figure 1b). Anti-GFP showed expression of each protein, however, the CD248-GFP band migrated at a lower molecular weight (~120 kDa) than previously reported (~175 kDa), or than expected with a GFP tag (~205 kDa),^17^ suggesting defects in glycosylation. Indeed, C-terminal tagging of CD248 has been shown to prevent cell surface expression.^18^ Therefore, CD248-GFP is most likely misfolded and we were unable to determine from this experiment whether CD248 binds MMRN2, this was addressed in further studies below.

To validate the CD93–MMRN2 interaction in endothelial cells, human umbilical vein endothelial cell (HUVEC) lysate was immunoprecipitated with mouse polyclonal MMRN2 antibodies or previously validated CD93 monoclonal antibody R139.^19, 20^ MMRN2 was enriched in CD93 immunoprecipitations (Figure 1c) and CD93 enriched in MMRN2 immunoprecipitations (Figure 1d).

### CLEC14A and CD93 bind a non-glycosylated coiled-coil region of MMRN2

**Figure 2:**
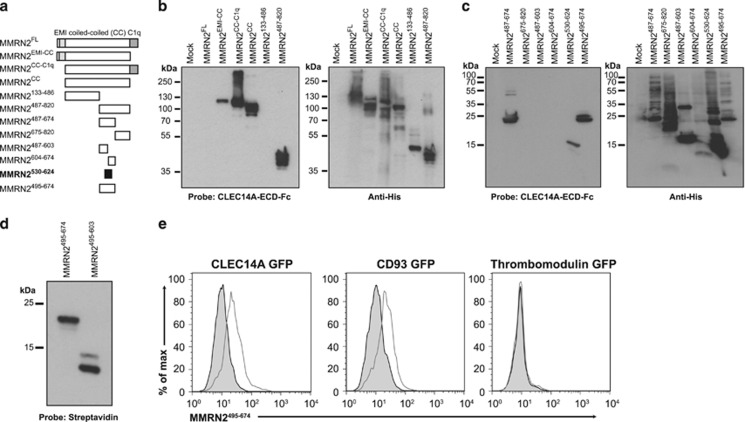
CLEC14A binds MMRN2 between residues 530 and 624. (**a**) Diagrams of MMRN2 truncation mutants, showing the elastin microfibril interface (EMI) domain, coiled-coil (CC) domain and complement related C1q domain. The minimal CLEC14A-binding fragment is highlighted in black. (**b** and **c**) HEK293T were transfected with truncation mutants, lysates were separated by SDS–PAGE under reducing conditions and far western blotted with CLEC14A-ECD-Fc and western blotted with His tag antibodies. (**b**) The smallest binding fragment was identified as MMRN2^487^^–^^820^, MMRN2^FL^ fails to bind CLEC14A-ECD-Fc. (**c**) Further truncation mutants were far western blotted revealing the smallest MMRN2 fragment binding CLEC14A is MMRN2^530^^–624^. (**d**) Purified MMRN2^495^^–674^ and MMRN2^495^^–603^ bind to streptavidin under reducing conditions after biotinylation. (**e**) Flow cytometry histograms of HEK293T transfected with GFP-tagged CTLD group 14 family members and stained with biotinylated MMRN2^495^^–674^ (grey line), confirming binding to CLEC14A and CD93 but not thrombomodulin. Streptavidin-PE alone was used as a control for background binding (grey shaded).

MMRN2 is comprised of three structural domains, an N-terminal EMI domain, a central coiled-coil domain and a C-terminal C1q domain.^21^ To characterize the CLEC14A-binding region, His tagged MMRN2 constructs were generated, each lacking major domains (Figure 2a). These included MMRN2^FL^, EMI and coiled-coil (MMRN2^EMI-CC^), coiled-coil and C1q (MMRN2^CC-C1q^), coiled-coil alone (MMRN2^CC^) and the coiled-coil halved (MMRN2^133^^–^^486^ and MMRN2^487^^–820^). The constructs were transfected into HEK293T and lysates probed with recombinant CLEC14A extracellular domain with an Fc tag (CLEC14A-ECD-Fc),^4^ binding occurred on all MMRN2 proteins except MMRN2^133^^–486^ and MMRN2^FL^ (Figure 2b). This is surprising as non-reduced MMRN2^FL^ does bind CLEC14A (Supplementary Figure S1). Despite this, the CLEC14A-binding region was clearly within MMRN2^487^^–820^.

To further characterize the CLEC14A-binding domain, MMRN2^487^^–820^ was divided in half revealing binding to MMRN2^487^^–674^ (Figure 2c). When further subdivided, binding occurred within MMRN2^530^^–624^ but not MMRN2^487^^–603^ or MMRN2^604^^–674^ constructs (Figure 2c). There exists a highly conserved region within this portion of MMRN2 (residues 588–620), suggesting a potentially evolutionary conserved CLEC14A-binding motif (Supplementary Figure S2). The non-binding fragment MMRN2^487^^–603^ terminates within this highly conserved region adding credence to this theory.

Due to low expression levels and the failure of MMRN2^530^^–624^ to be efficiently expressed and purified, this fragment was not pursued further. Work focussed on the second smallest fragment MMRN2^487^^–674^. As this fragment forms disulphide-linked high molecular weight complexes, which could interfere in downstream assays, the two N-terminal cysteine residues were removed. The resulting MMRN2^495^^–674^ construct maintained its ability to bind CLEC14A (Supplementary Figure S3).

To explore whether this binding domain existed in mice, the corresponding regions in mouse MMRN2 (495–678) were expressed in HEK293T and mouse CLEC14A-ECD-Fc far western blotting revealed positive binding (Supplementary Figure S4). The human MMRN2^495^^–674^ fragment along with the non-CLEC14A-binding fragment MMRN2^495^^–603^ was expressed in *E. coli* with a BirA tag for specific biotinylation.^22^ Both biotinylated proteins bound streptavidin (Figure 2d), and biotinylated MMRN2^495^^–674^ bound to cell surface expressed CLEC14A and CD93 but not thrombomodulin, confirming CLEC14A and CD93 bind to a similar MMRN2 region (Figure 2e).

### MMRN2 binding is dependent on the CLEC14A-CTLD

**Figure 3:**
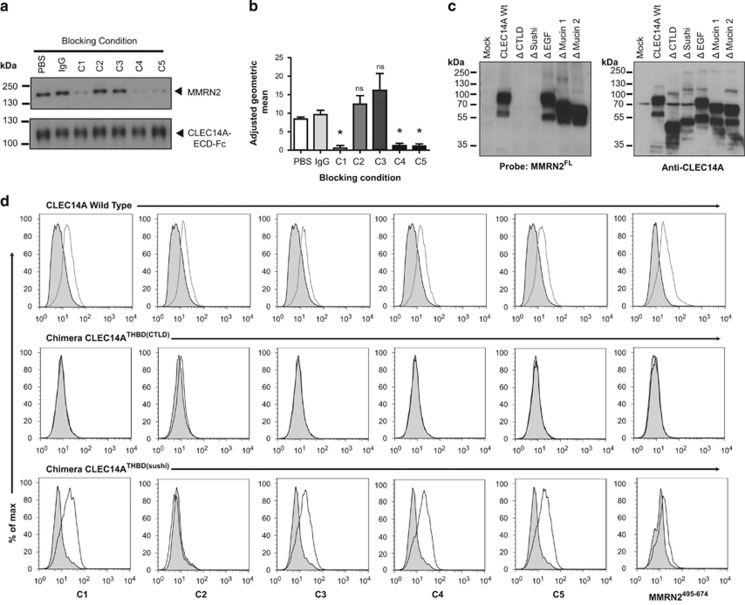
MMRN2 binds to CLEC14A in the C-type lectin domain. (**a**) CLEC14A-ECD-Fc pull-downs were pre-incubated with either PBS, mouse control IgG, or CLEC14A monoclonal antibodies C1–5 and used to pull down MMRN2^FL^ from HEK293T-transfected lysates. C1, C4 and C5 blocked MMRN2 enrichment, whereas C2 and C3 did not. (**b**) Flow cytometry analysis of CLEC14A-ECD-Fc binding to HUVEC surface. CLEC14A-ECD-Fc was pre-incubated with the same blocking conditions as in pull-downs. C1, C4 and C5 significantly blocked CLEC14A-ECD-Fc binding to HUVEC surface. (*P<0.05 Mann–Whitney test *n*=4) error bars represent standard error mean (s.e.m.). (**c**) HEK293T transfected with CLEC14A domain deletions were lysed and far western blotted with MMRN2^FL^ under non-reduced conditions. Upon detection of the MMRN2^FL^ His tag, MMRN2 binding was observed in all mutants except those lacking the CTLD or sushi domains. Probing with anti-CLEC14A antibodies was included to show expression of each mutant protein. (**d**) Flow cytometry analysis of HEK293T transfected with CLEC14A wild-type (wt) with a GFP tag, chimera 1 CLEC14A^THBD(CTLD)^ or chimera 2 CLEC14A^THBD(sushi)^. All of the CLEC14A monoclonal antibodies and MMRN2^495^^–^^674^ bound to CLEC14A wt. None of the CLEC14A monoclonal antibodies nor the MMRN2^495^^–674^ fragment bound to chimera 1 CLEC14A^THBD(CTLD)^ except modest binding with C2. All antibodies except C2 bound to chimera 2 CLEC14A^THBD(sushi)^. The MMRN2^495^^–674^ fragment could also bind chimera 2 CLEC14A^THBD(sushi)^.

Our previously described CLEC14A–MMRN2 blocking antibody C4 and the non-blocking antibody C2 provide useful tools in determining important CLEC14A-binding regions.^3^ To examine whether monoclonals C1, C3 or C5 also exhibited blocking effects, CLEC14A-ECD-Fc pull-down assays blocked with control IgG or C1–C5 were performed on HEK293T MMRN2^FL^ overexpressed lysates. Blocking was observed by C1, C4 and C5 antibodies but not C2 or C3 (Figure 3a). C1, C4 and C5 could also block CLEC14A-ECD-Fc from binding to the HUVEC surface (Figure 3b). These antibodies only bind in flow cytometry and not western blots under reducing conditions, offering ideal tools for probing the natural conformational folding of CLEC14A.

To establish which CLEC14A domain binds MMRN2, CLEC14A deletion constructs and far western blotting was used. MMRN2^FL^ failed to bind CLEC14A lacking the CTLD or sushi domain (Figure 3c). This could be due to MMRN2 binding being dependent on both domains, or CLEC14A does not fold correctly when lacking one of these domains. To explore the latter, chimeric CLEC14A constructs were generated using the CTLD of the non-MMRN2 binding thrombomodulin (denoted CLEC14A^THBD(CTLD)^) and the sushi of thrombomodulin (CLEC14A^THBD(sushi)^) inserted into full-length CLEC14A-GFP.

Flow cytometry showed lack of binding of all CLEC14A antibodies to CLEC14A^THBD(CTLD)^ except moderate C2 binding. CLEC14A^THBD(sushi)^ was able to bind all CLEC14A antibodies except C2 (Figure 3d). This confirmed that the chimeras were correctly folded and present on the cell surface and showed that binding epitopes for all anti-CLEC14A antibodies were within the CTLD except for C2. Similarly, MMRN2^495^^–674^ could bind CLEC14A^THBD(sushi)^ but not CLEC14A^THBD(CTLD)^, confirming that the CLEC14A-CTLD is required for MMRN2 binding (Figure 3d).

### CD248 binds to a separate region of MMRN2 from CLEC14A and CD93

**Figure 4:**
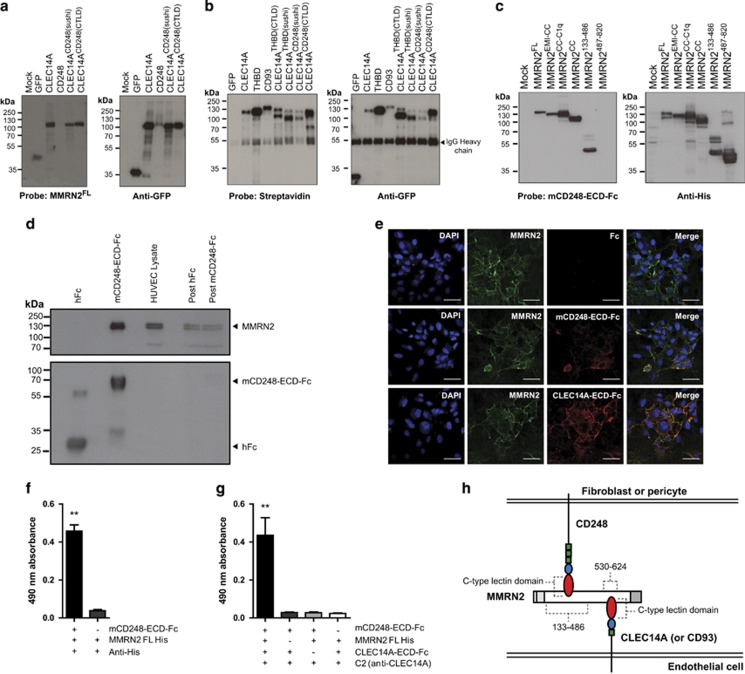
CD248 binds to MMRN2 in a distinct region from CLEC14A and CD93 binding. (**a**) HEK293T transfected with GFP, CLEC14A-GFP, CD248-GFP, and chimeras CLEC14A^CD248(sushi)^ and CLEC14A^CD248(CTLD)^ were lysed and far western blotted with MMRN2^FL^ and western blotted with anti-GFP. MMRN2^FL^ binds to CLEC14A and both chimeras CLEC14A^CD248(sushi)^ and CLEC14A^CD248(CTLD)^ but not CD248-GFP or GFP alone. (**b**) Immunoprecipitations of GFP-tagged proteins after cell surface biotinylation. CLEC14A, THBD, CD93 and all four CLEC14A chimeras bind to streptavidin showing they are cell surface expressed. GFP alone was included to demonstrate intracellular proteins are not cell surface biotinylated. (**c**) MMRN2 truncation mutants were transfected into HEK293T and lysates under reducing conditions were far western blotted with mCD248-ECD-Fc, revealing binding to minimal fragment MMRN2^133^^–486^. His tag western blot confirmed expression of each MMRN2 protein fragment. (**d**) mCD248-ECD-Fc pull-downs from HUVEC lysates resulted in enrichment of MMRN2 compared with hFc control. (**e**) Immunofluorescence analysis of HUVEC stained with MMRN2 mouse polyclonal antibodies, hFc, mCD248-ECD-Fc or CLEC14A-ECD-Fc. MMRN2 antibody staining partially co-localizes with CD248-ECD-Fc and CLEC14A-ECD-Fc binding; scale bar, 40 μm. (**f**) ELISAs of mCD248-ECD-Fc bound to plate capturing MMRN2 FL His, (**P<0.01 Mann–Whitney test, *n*=5) error bars represent s.e.m. (**g**) ELISAs of mCD248-ECD-Fc capturing MMRN2 FL His and then binding by CLEC14A-ECD-Fc detected by anti-CLEC14A antibody C2. (**P<0.01 Mann–Whitney test, *n*=5) error bars, s.e.m. (**h**) Diagram of CLEC14A or CD93 expressed by endothelial cells binding to MMRN2 in the ECM, which in turn is bound by CD248 expressed by fibroblasts or vasculature associated pericytes. CD248, CLEC14A and CD93 binding is due to the CTLD, CD248 binds MMRN2 in the region 133–486, whereas CLEC14A binds in the region 530–624.

To determine whether the CD248-CTLD binds MMRN2 in a correctly folded and cell surface expressed form, the domain was replaced in CLEC14A to create chimera CLEC14A^CD248(CTLD)^. This was expressed in HEK293T and lysates far western blotted under non-reducing conditions revealed binding by MMRN2^FL^ (Figure 4a). To test whether the sushi domain of CD248 was sufficient to confer correct folding of CLEC14A-CTLD the chimera CLEC14A^CD248(sushi)^ was generated, this also bound MMRN2^FL^.

To ensure GFP-tagged wild-type (wt) and chimeric proteins were expressed at the cell surface, transfected HEK293T were cell surface biotinylated before anti-GFP immunoprecipitation. Probing with streptavidin-horse radish peroxidase (HRP) confirmed CLEC14A, CD93, thrombomodulin and chimeras were expressed on the cell surface (Figure 4b).

To determine where CD248 binds MMRN2, truncation mutants were transfected into HEK293T and lysates subjected to far western blot analysis. Mouse CD248-ECD-Fc that had been previously shown to bind human endothelial ECM was used as a probe.^11^ CD248-ECD-Fc bound to MMRN2^133^^–486^, a completely distinct region than required for CLEC14A or CD93 binding (Figure 4c). To test whether mCD248-ECD-Fc could bind MMRN2 from HUVEC, pull-down assays were performed on lysates, revealing enrichment of MMRN2 (Figure 4d).

To determine whether previous mCD248-ECD-Fc ECM staining experiments were due to binding MMRN2,^11^ mCD248-ECD-Fc and polyclonal MMRN2 antibodies were used to stain cultured HUVEC. MMRN2 staining revealed fibrous meshes in the ECM partially co-localizing with mCD248-ECD-Fc or CLEC14A-ECD-Fc binding but not hFc alone (Figure 4e).

To determine whether CLEC14A and CD248 can bind MMRN2 simultaneously, a sandwich ELISA (enzyme-linked immunosorbent assay) approach was taken. This showed CD248 could capture MMRN2 (Figure 4f), and CD248 could also capture MMRN2, which subsequently bound CLEC14A, confirming these proteins do not compete for binding with MMRN2 (Figure 4g). This suggests CLEC14A or CD93 expressed by endothelial cells can bind MMRN2 at the same time as CD248 expressed by fibroblasts or pericytes (Figure 4h). Descriptions of each MMRN2 truncation and which CTLD group 14 members bind are summarized in Supplementary Table 1.

**Figure 5:**
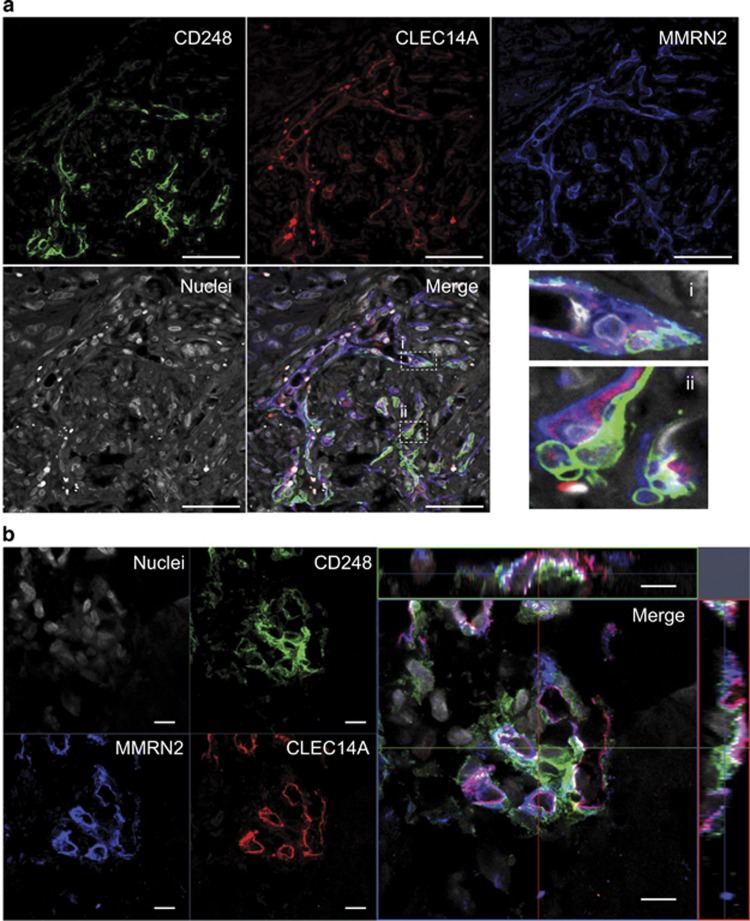
CLEC14A–MMRN2–CD248 co-localization can be observed in human pancreatic cancer. Human pancreatic cancer sections were stained with antibodies against CD248 (green), CLEC14A (red) and MMRN2 (blue), Hoescht was used to visualize nuclei (grey). (**a**) CD248-positive cells (likely pericytes) are in close proximity to CLEC14A- and MMRN2-positive endothelial cells. This revealed co-localization of CLEC14A and MMRN2 in association with CD248-positive cells. Dotted lines highlight areas that have been enlarged (i and ii). Scale bars, 50 μm. (**b**) Partial co-localization of CLEC14A and MMRN2 where they meet CD248-positive cells. Co-localization of all three proteins is indicated in white. The merged image displays orthogonal views in *xz* and *yz*. Scale bars, 10 μm.

To determine whether the CLEC14A–MMRN2–CD248 interaction could be observed in human cancer, pancreatic tumours were stained with antibodies against each protein, revealing separate CLEC14A and MMRN2 expression from CD248. In some areas, co-localization of all three proteins can be seen at the interface between CLEC14A-positive endothelial cells and CD248-positive cells, likely to be pericytes. (Figure 5).

### MMRN2 binding is dependent on a CTLD long-loop region in CLEC14A and CD93

**Figure 6:**
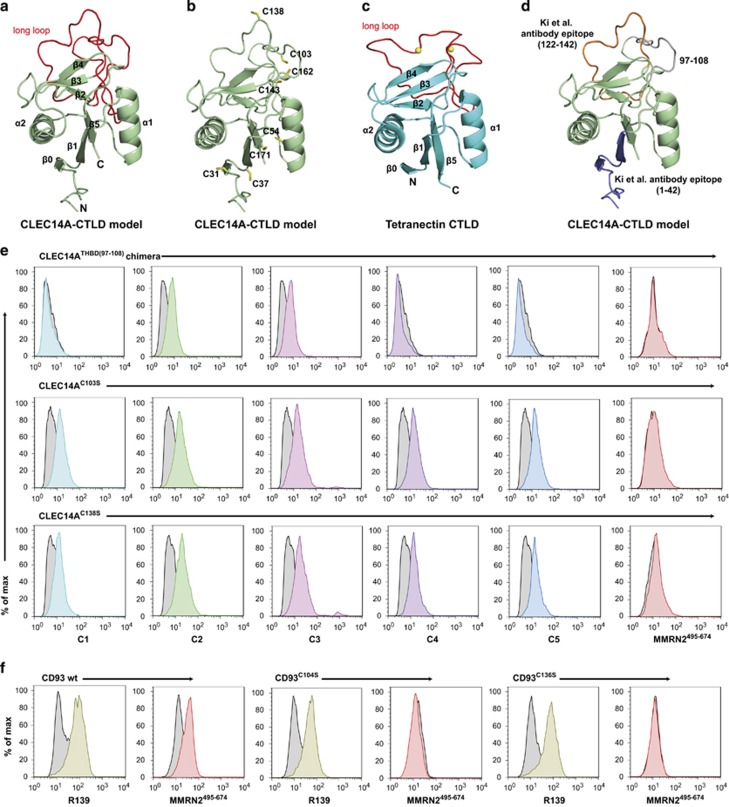
CLEC14A and CD93 bind MMRN2 in long-loop region of the CTLD. (**a**) CLEC14A-CTLD iTASSER generated molecular model, displaying long-loop region in red and numbered α-helices and β-sheets. (**b**) CLEC14A-CTLD model displaying predicted arrangement of cysteine residues. Cysteines C31, C37, C54, C143, C162 and C171 are canonical CTLD fold cysteines and are in close proximity to form disulphide bonds. C103 and C138 are non-canonical cysteines present in the long-loop region. (**c**) Solved crystal structure of tetranectin CTLD (1TN3), displaying long-loop region in red and numbered α-helices and β-sheets. (**d**) CLEC14A-CTLD model with predicted antibody epitopes 1–42 (showing 21–42 in the model, blue), 122–142 (orange) and region 97–108 (grey). (**e**) Flow cytometry analysis of HEK293T transfected with CLEC14A^THBD(97^^–108)^, CLEC14A^C103S^ or CLEC14A^C138S^. Only C2 and C3 bind to CLEC14A^THBD(97^^–108)^, all C1–5 bind CLEC14A^C103S^ and CLEC14A^C138S^ all CLEC14A mutant proteins fail to bind MMRN2^495^^–674^. (**f**) Flow cytometry analysis of HEK239T transfected with CD93 wt, CD93^C104S^ or CD93^C136S^. All proteins bind R139 antibody showing correct conformational folding and presence at the cell surface, although both CD93^C104S^ and CD93^C136S^ fail to bind MMRN2^495^^–674^.

To visualize the three-dimensional orientation of the CLEC14A-CTLD and potential MMRN2 recognition surfaces, a predicted molecular model of the CLEC14A-CTLD was generated using the iTASSER server.^23^ This model exhibited characteristics of the CTLD fold, including a ‘loop in a loop’ structure with a hydrophobic core^24^ (Figure 6a), and revealed the close proximity of six cysteine residues that are canonical in CTLDs suggesting disulphide bond formation (Figure 6b). There are also two non-canonical cysteines within the long-loop region that are distal from each other (C103 and C138). The CLEC14A-CTLD model displays a similar overall structure to the crystal structure of human tetranectin^25^ (Figure 6c).

A previous study demonstrated that CTLD-specific CLEC14A antibodies had similar anti-angiogenic effects as observed with our C4 antibody, we hypothesized that these may block the CLEC14A–MMRN2 interaction.^26^ These CTLD-specific antibodies have been described to bind epitopes spanning amino acids 1–42 or 122–142 of CLEC14A.^27^ These regions were mapped onto the predicted CLEC14A-CTLD model, revealing 1–42 is proximal to the sushi domain boundary and 122–142 is on the long loop. There also existed another region (97–108), which was semi-conserved in CD93 and part of the predicted long loop (Figure 6d and Supplementary Figure S5). To test whether epitopes for our antibodies or regions important for MMRN2 binding were within these regions, CLEC14A chimeras were generated by swapping with corresponding regions of thrombomodulin. CLEC14A^THBD(1^^–^^42)^ and CLEC14A^THBD(122^^–142)^ chimeras failed to bind antibodies C1–C5 suggesting they were incorrectly folded, and were not used for further experiments (data not shown). In contrast, the CLEC14A^THBD(97^^–108)^ mutant could bind C2 and C3 but not the CLEC14A–MMRN2 blocking antibodies C1, C4 or C5, indicating the binding epitopes for these antibodies are within this region (Figure 6e). MMRN2^495^^–674^ also failed to bind CLEC14A^THBD(97^^–108)^ as expected. The 97–108 region contained the amino acids 97ERRRSHCTLENE108, to test whether the non-canonical cysteine (C103) within this sequence forms disulphide bonds that are important for MMRN2 binding, the mutant CLEC14A^C103S^ was generated along with the other non-canonical long-loop cysteine (CLEC14A^C138S^). These mutants could bind all CLEC14A monoclonals C1–C5 suggesting they were correctly folded. However, they failed to bind MMRN2^495^^–674^, highlighting the importance of these residues for CLEC14A–MMRN2 interactions (Figure 6e).

As CD93 also contains two non-canonical cysteines in the predicted long-loop region, the mutants CD93^C104S^ and CD93^C136S^ were generated. The monoclonal R139 anti-CD93 antibody is conformation-sensitive and was used to validate correct folding and cell surface expression of CD93 mutants. Both cysteine mutants along with CD93 wild-type (wt) could bind R139 but mutants failed to bind MMRN2^495^^–674^ (Figure 6f). This confirmed the necessity of these cysteines for CD93–MMRN2 interactions as observed for CLEC14A–MMRN2.

### The CLEC14A and CD93 binding fragment of MMRN2 inhibits angiogenesis *in vitro*

**Figure 7:**
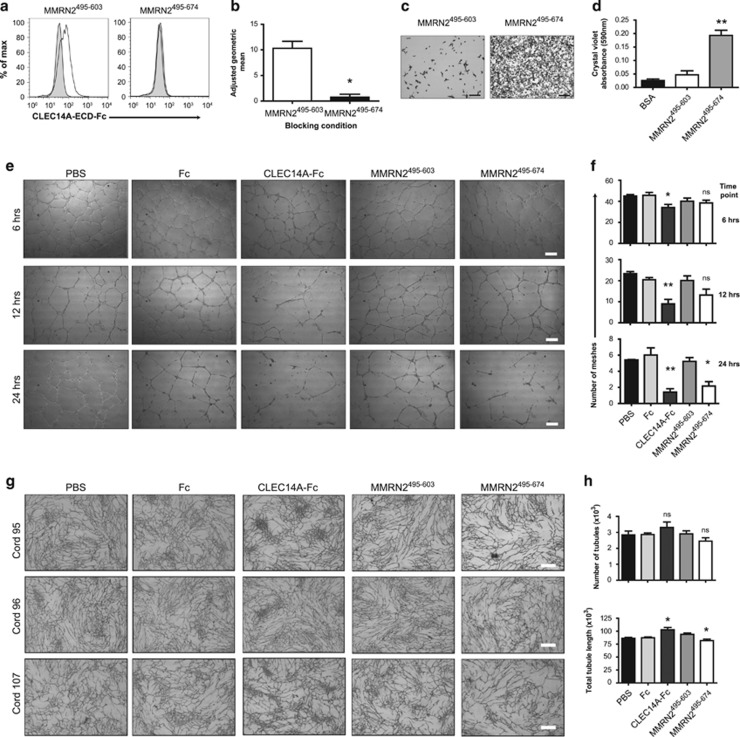
MMRN2^495^^–674^ inhibits angiogenesis *in vitro*. (**a**) Representative flow cytometry histograms of CLEC14A-ECD-Fc pre-incubated with MMRN2^495^^–674^ or MMRN2^495^^–603^ and then used to stain HUVEC surface, hFc used as isotype control. (**b**) MMRN2^495^^–674^ significantly blocked cell surface binding. (*P<0.05 Mann–Whitney test *n*=4). (**c**) Representative images of MMRN2^495^^–603^ and MMRN2^495^^–674^ coated plates with adherent HUVEC stained with crystal violet; scale bar, 150 μm. (**d**) HUVEC adhered significantly more to MMRN2^495^^–674^ compared with MMRN2^495^^–603^ or bovine serum albumin (BSA; **P<0.01 Mann–Whitney test, *n*=6). (**e**) HUVEC were added to Matrigel and treated with PBS, hFc, CLEC14A-ECD-Fc (CLEC14A-Fc) MMRN2^495^^–603^ or MMRN2^495^^–674^. Representative images from 6, 12 and 24 h post Matrigel plating from one of three independent experiments; scale bar, 100 μm. (**f**) Quantification of number of meshes at 6, 12 and 24 h time points, average values from eight fields of view from three independent experiments, (*P<0.05, **P<0.01 unpaired *t*-test, *n*=3, error bars represent s.e.m.). (**g**) Representative images of HUVEC-fibroblast co-culture assay from three different umbilical cords. Scale bar, 800 μm. (**h**) Quantification of number of tubules and total tubule length, from averages of four fields of view from three independent experiments. (*P<0.05 unpaired *t*-test, *n*=3, error bars represent s.e.m.).

To test whether blocking CLEC14A and CD93 interacting with MMRN2 can affect angiogenesis, the MMRN2^495^^–674^ fragment and the non-binding MMRN2^495^^–603^ fragment were expressed and purified from *E. coli* (Supplementary Figure S6). The same experiment as Figure 3b was performed testing blocking function of MMRN2^495^^–674^ or MMRN2^495^^–603^ on CLEC14A-ECD-Fc binding to HUVEC. This resulted in significant blocking with MMRN2^495–674^ (Figures 7a and b).

MMRN2 was previously shown to increase HUVEC adherence.^28^ To test whether the MMRN2^495^^–674^ fragment could also increase adherence, the plates were coated with MMRN2^495^^–674^, MMRN2^495^^–603^ or BSA control, resulting in HUVEC adhering to MMRN2^495^^–674^ but not MMRN2^495^^–603^ or BSA (Figures 7c and d).

The MMRN2^495^^–674^ and MMRN2^495^^–603^ fragments were next examined in angiogenesis assays. As we have previously shown, CLEC14A-ECD-Fc has anti-angiogenenic effects,^4^ this was included in all assays as a positive control along with human IgG Fc alone to account for effects of the Fc tag. Recombinant proteins were added to HUVEC in Matrigel tube formation assays resulting in significant decreases in tubule mesh formation with CLEC14A-ECD-Fc and MMRN2^495^^–674^ compared with Fc and MMRN2^495^^–603^ respectively (Figures 7e and f). Recombinant proteins were then tested in the organotypic human fibroblast-HUVEC co-culture assay,^29^ resulting in modest reductions in the number of tubules and total tubule length when treated with MMRN2^495^^–674^, but in this case not when treated with CLEC14A-ECD-Fc (Figures 7g and h). Intriguingly, CLEC14A-ECD-Fc treatments in co-cultures induced formation of knot-like areas with high density of tubules.

### The CLEC14A and CD93 binding fragment of MMRN2 reduces tumour growth

**Figure 8:**
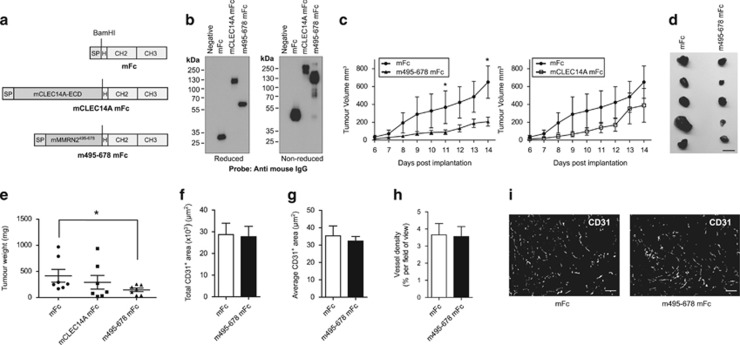
Mouse MMRN2^495^^–678^ reduces tumour growth. (**a**) Diagrams of constructs used to transduce Lewis lung carcinoma cells. Mouse hinge region (H), Constant heavy chain 2 and 3 (CH2 and CH3) were fused to mouse CLEC14A signal peptide (SP) to allow secretion of mouse Fc (mFc). Mouse Fc was also fused to mouse CLEC14A-ECD (mC14A mFc) and mouse MMRN2^495^^–678^ (m495-678 mFc). (**b**) Western blots detecting mouse Fc tag in conditioned media of LLC transduced with each fusion gene. All proteins are secreted and form dimers under non-reduced conditions. (**c**) Tumour volume was measured from days 6 to 14 post implantation. The same mFc control group are plotted on each graph to allow comparison with m495-678 mFc or mC14A mFc (*P<0.05 Mann–Whitney test, *n*=7). (**d**) Representative image of tumours from mFc and m495-678 mFc LLC post excision. Scale bar, 10 mm. (**e**) End-point tumour weight of mFc, mC14A mFc and m495-678 mFc. (*P<0.05 Mann–Whitney test, *n*=7). Error bars represent s.e.m. (**f**) Quantification of total area of CD31 immunofluorescence staining from mFc and m495-678 mFc tumour sections. (**g**) Average area stained positively for CD31. (**h**) Vessel density as a percentage of CD31 staining per field of view. (**f**–**h**) Averages of five fields of view from each tumour (*n*=3). (**i**) Representative images of CD31 immunofluorescence staining of mFc and m495-678. Scale bar, 100 μm.

To test whether disrupting CLEC14A and CD93 interactions had an effect on tumour growth *in vivo*, the mouse MMRN2^495^^–678^ fragment and the mouse CLEC14A-ECD were fused to a mouse IgG Fc tag. These constructs included the signal peptide of mouse CLEC14A (mCLEC14A) to allow secretion along with murine Fc as a control (Figure 8a). Lewis lung carcinoma (LLC) cells were separately lentivirally transduced with these constructs, achieving greater than 90% transduction efficiency (Supplementary Figure S7). This Fc-fusion strategy was utilized to increase serum half-life and allow these proteins to be expressed at local sites of neo-angiogenesis by cells of mouse origin. Western blots of conditioned media confirmed secretion (Figure 8b). Transduced LLC cells were shown to have no differences in proliferation *in vitro* (Supplementary Figure S8), and were subcutaneously injected into the flanks of C57BL/6 mice. Tumour growth was monitored by daily calliper measurements, revealing growth delays in mMMRN2^495^^–^^678^mFc LLC tumours compared with mFc (Figure 8c). End tumour weights revealed a significant reduction in mMMRN2^495^^–678^mFc expressing LLC (Figures 8d and e). There was no significant difference in the weights or growth rates of mCLEC14A-ECD-mFc expressing tumours. Immunofluorescence analysis of CD31-positive vessels revealed no clear differences between mFc and mMMRN2^495^^–678^mFc (Figures 8f–i), although due to the defects observed in angiogenesis assays, it is possible that these vessels while present, are non-functional.

## Discussion

The CTLD group 14 family are important emerging molecules in tumour angiogenesis. Our present study has demonstrated CD93 and CD248 as being able to bind the CLEC14A ECM ligand MMRN2. These interactions have been dissected and found to involve a predicted long loop in the CTLD of CLEC14A and CD93, and regions of MMRN2 within its coiled-coil domain. The CLEC14A and CD93 binding fragment of MMRN2 had anti-angiogenic effects presumably by disrupting normal CLEC14A and CD93 function.

MMRN2 binding is the first description of an extracellular ligand for CD93 and explains previous observations, such as the ability of CD93-CTLD-Fc to stain endothelium in human tonsils and CD93 roles in cell adhesion.^30, 31^ CD93 is also important in endothelial migration and tube formation, CD93-deficient mice exhibit angiogenesis defects in tumour models phenocopying observations made for CLEC14A.^2, 3, 5, 7, 32^ We hypothesize that such effects are potentially due to no longer being present to bind MMRN2.

The CD248–MMRN2 interaction also offers explanations to previous findings. The CD248-ECD has been used as a binding probe in immunofluorescence studies on mouse tissues and cultured cells,^11^ revealing characteristic ECM staining only occurring on endothelial cells, likely dependent on MMRN2. CD248–MMRN2 interactions occur on a separate region from CLEC14A and CD93 binding, suggesting endothelial expressed CTLD group 14 members can bind to MMRN2 simultaneously with CD248 expressed by other cell types such as pericytes or fibroblasts. Indeed, this is the case for CLEC14A–MMRN2–CD248 interactions in pancreatic cancer. This offers a scenario where MMRN2 acts as an ‘extracellular glue’ between both cell types in vessel formation and maturation. This adds to the list of ECM proteins along with collagens I and IV and fibronectin already described as potential CD248 ligands.^33^

We have dissected the molecular characteristics of these interactions, revealing a predicted long-loop region within both CLEC14A and CD93 CTLDs where two conserved cysteine residues are essential for MMRN2 interactions. These cysteines are likely to be important in the local conformation of the long-loop region and disulphide bond formation may be important. As the conformation-sensitive CLEC14A antibodies bind to both of these cysteine mutants, disruptions in the folding of the whole CLEC14A molecule have been ruled out. Nevertheless, although these residues are not important for antibody binding, they are vital for binding to MMRN2.

The relevance of MMRN2 in angiogenesis has been previously demonstrated, two studies describe it as an angiostatic molecule, acting by sequestering VEGF-A.^28, 34^ However, our studies and those by Zanivan *et al.*^10^ describe MMRN2 as a pro-angiogenic molecule binding to cell surface proteins. These conflicting roles could be context dependent, where CLEC14A, CD93 and CD248 interactions are separate from those of VEGF-A. Our observations of HUVEC adhering to MMRN2^495^^–^^674^ could explain the calcium-independent adhesion described for MMRN2^FL^, with CLEC14A, CD93 or both eliciting this adhesive effect.^28^

CLEC14A and CD93 may have similar roles in ECM interactions, although it is unclear whether these interactions have distinct signalling outcomes or compensatory roles. As CD93 is expressed by other cell types such as haematopoietic cells and platelets,^35^ these are also likely to bind MMRN2 and the endothelial ECM. Future studies will shed light on the roles of CLEC14A, CD93 and CD248 and the signalling of these understudied molecules.

## Materials and methods

### Antibodies and reagents

Antibodies: anti-CLEC14A mouse monoclonals C1–C5 generated in our laboratory,^3^ anti-CLEC14A sheep polyclonal (R&D Systems, Abingdon, UK, #AF4968), mouse anti-His clone AD1.1 (R&D Systems, #MAB050), anti-GFP mouse clone 3E1 (Cancer Research UK), anti-MMRN2 mouse polyclonal (Abnova/Novus Biologicals, Abingdon, UK, #H00079812-B01P), anti-MMRN2 rabbit polyclonal (Abcam, Cambridge, UK, #ab171314), anti-CD93 mouse clone R139 (Thermo Fisher Scientific, Rugby, UK, #14-0939), anti-CD93 goat polyclonal (R&D Systems, #AF2379), anti-CD248 mouse clone B1.35 (hybridoma supernatants), anti-CD31 mouse clone JC70A (Dako, Cambridge, UK, #M0823), anti-Tubulin mouse clone DM1A (Sigma-Aldrich, Gillingham, UK, #T9026), anti-fibronectin sheep polyclonal (R&D Systems, #AF1918), anti-CD31 rat clone MEC13.3 (BD Bioscience, Oxford, UK, #565629), mouse IgG isotype control (Thermo Fisher Scientific, #10400C), human IgG Fc (Bethyl Laboratories, Cambridge, UK, #P80-104) (sodium azide removed by dialysis in phosphate-buffered saline (PBS)), anti-human IgG Fc HRP conjugated (Sigma-Aldrich, #A0170), anti-mouse HRP (Dako, #P0447), anti-sheep HRP (R&D Systems, #HAF016), anti-goat HRP (Dako, #P0449), streptavidin HRP (GE Healthcare, Amersham, UK, #RPN1231) anti-mouse alexafluor-555 (Thermo Fisher Scientific, #A21425), anti-human IgG alexafluor-555 (Thermo Fisher Scientific, #A21433), anti-mouse alexafluor-488 (Thermo Fisher Scientific, #A11001), anti-rabbit alexafluor-647 (Thermo Fisher Scientific, #A31573), anti-sheep alexafluor-546 (Thermo Fisher Scientific, #A21098), anti-hFc FITC conjugated (Sigma-Aldrich, #F9512), streptavidin R-phycoerthrin (PE) conjugate (Thermo Fisher Scientific, #S-866), fibronectin (Sigma-Aldrich, #F2006). CD248-ECD-Fc as used.^11^

### Plasmid construction

All CTLD 14 members, mutants and chimeras inserted between EcoRI in pEGFPN1, using Gibson assembly according to manufacturer (New England Biolabs, Hitchin, UK), using PCR products amplified with the following primers; CLEC14A-forward 5′-GATCTCGAGCTCAAGCTTCGATGAGGCCGGCGTTCGCC-3′, CLEC14A-reverse 5′-TACCGTCGACTGCAGTGCATCACTAGAGCCAAG-3′, CD93-forward 5′-CGAGCTCAAGCTTCGATGGCCACCTCCATGGGC-3′, CD93-reverse 5′-TACCGTCGACTGCAGGCAGTCTGTCCCAGGTGTCG-3′, THBD-forward 5′-CGAGCTCAAGCTTCGATGCTTGGGGTCCTGGTC-3′, THBD-reverse 5′-TACCGTCGACTGCAGGAGTCTCTGCGGCGTCCG-3′, CD248-forward 5′-CGAGCTCAAGCTTCGATGCTGCTGCGCCTGTTG-3′, CD248-reverse 5′-TACCGTCGACTGCAGTCACACGCTGGTTCTGCAG-3′. For chimeras, two PCR products or more were Gibson assembled using; (CLEC14A^THBD(CTLD)^; THBD-forward and THBD-CTLD fused to CLEC14A-sushi-reverse 5′-CTCAAACTGGAACTCGCAGAGGAAGCC-3′, THBD-CTLD fused to CLEC14A-sushi-forward 5′-GCGAGTTCCAGTTTGAGGTCTTGTGTC-3′ and CLEC14A-reverse). (CLEC14A^THBD(sushi)^; CLEC14A-forward and CLEC14A-CTLD fused to THBD-sushi-reverse 5′-TACCGTCGACTGCAGTGCATCACTAGAGCCAAG-3′, CLEC14A-CTLD fused to THBD-sushi-forward 5′-GTGCAAGTACCACTTCCCAGCCACCTGCAGGC-3′ and THBD-sushi fused to CLEC14A-EGF-reverse 5′-TCCCGGGGCAAGCGCCCGGCGCCTCCCT-3′, THBD-sushi fused to CLEC14A-EGF-forward 5′-GCCGGGCGCTTGCCCCGGGAGGTACCTC-3′ and CLEC14A-reverse). (CLEC14A^C103S^; CLEC14A-forward and CLEC14A^C103S^-reverse 5′-CTCGTTCTCCAGGGTTGAGTGGGAACGCCTGCGCTC-3′, CLEC14A^C103S^-forward 5′-GAGCGCAGGCGTTCCCACTCAACCCTGGAGAACGAG-3′ and CLEC14A-reverse). (CLEC14A^C138S^; CLEC14A-forward and CLEC14A^C138S^-reverse 5′-CGCGCATCTCCGCGCGGTGGAGGAGCGTTGGGGCTCCTC-3′, CLEC14A^C138S^-forward 5′-GAGGAGCCCCAACGCTCCTCCACCGCGCGGAGATGCGCG-3′ and CLEC14A-reverse). (CLEC14A^CD248(sushi)^; CLEC14A-forward and CLEC14A-CTLD fused to CD248-sushi-reverse 5′-CCTCGAAGCCGTACTTGCACAGGTAGCCGTTGGC-3′, CLEC14A-CTLD fused to CD248-sushi-forward 5′-GTGCAAGTACGGCTTCGAGGGCGCCTGC-3′ and CD248-sushi fused to CLEC14A-EGF-reverse 5′-TCCCGGGGCAGCCAGTCCCCAGGCACAGG-3′, CD248-sushi fused to CLEC14A-EGF-forward 5′-GGGGACTGGCTGCCCCGGGAGGTACCTC-3′ and CLEC14A-reverse). (CLEC14A^CD248(CTLD)^; CD248-forward and CD248-CTLD fused to CLEC14A-sushi-reverse 5′-CTCAAACTGAAACTGGCACAGGTAGCCG-3′, CD248-CTLD fused to CLEC14A-sushi-forward 5′-GCCAGTTTCAGTTTGAGGTCTTGTGTC-3′ and CLEC14A-reverse).

MMRN2 fragments were amplified using; (MMRN2^EMI-CC^; MMRN2^FL^-forward 5′-CCGGACCGGTCAGGCTTCCAGTACTAGCC-3′ and MMRN2^820^-reverse 5′-CTACTAGGTACCCCAGAGCGCCGCGCCC-3′). (MMRN2^CC-C1q^; MMRN2^133^-forward 5′-CCGGACCGGTGATTCCATGGCAATCCCTGA-3′ and MMRN2^FL^-reverse 5′-CGGGGTACCGGTCTTAAACATCAGGAAGC-3′). (MMRN2^CC^; MMRN2^133^-forward and MMRN2^820^-reverse). (MMRN2^133^^–486^; MMRN2^133^-forward and MMRN2^486^-reverse 5′-CTACTAGGTACCCTTGATGAGGTCGGCATGG-3′). (MMRN2^487^^–820^; MMRN2^487^-forward 5′-CCGGACCGGTTACGTGAAGGACTGCAATTG-3′ and MMRN2^820^-reverse), (MMRN2^487^^–674^; MMRN2^487^-forward and MMRN2^674^-reverse 5′-CTACTAGGTACCCGGCCGCGGGGGCTCCG-3′) (MMRN2^675^^–820^; MMRN2^675^-forward 5′-CCGGACCGGTGCAGAGCACCTGGAGCC-3′ and MMRN2^820^-reverse) (MMRN2^487^^–603^; MMRN2^487^-forward and MMRN2^603^-reverse 5′-CTACTAGGTACCCGCGTCCTCCAGCAGGG-3′) (MMRN2^604^^–674^; MMRN2^604^-forward 5′-CCGGACCGGTCTGCGGCACGAGGCGGTG-3′ and MMRN2^674^-reverse) (MMRN2^530^^–624^; MMRN2^530^-forward 5′-CCGGACCGGTGGCTCCTCCCTGCAGGCC-3′ and MMRN2^624^-reverse 5′-CTACTAGGTACCCTCAGACATCTCCTCCAGC-3′) (MMRN2^495^^–674^; MMRN2^495^-forward 5′-TAGTAGACCGGTCAGAAGCTCTATTTAGACCTG-3′ and MMRN2^674^-reverse). (Mouse MMRN2^495^^–678^; mMMRN2^495^-forward 5′-CCGGACCGGTCAAAGGGTCAACTCTGACGTG-3′ and mMMRN2^678^-reverse 5′-CTACTAGGTACCCAACTGTGGGTGCTGCTCC-3′). AgeI and KpnI digested PCR products were ligated into pHL-Avitag3 containing; N-terminal signal peptide (SP), C-terminal BirA and His tag (avitag).^36^

Codon optimized MMRN2^495^^–674^ and MMRN2^495^^–603^ DNA was synthesized (IDT Technologies, Leuven, Belgium) with ends complementary to pET23a vector and Gibson assembled in between NdeI and NotI. The BirA sequence 5′-GGTGGTGGTCTGAACGATATTTTTGAAGCTCAGAAAATCGAATGG-3′ was used.

Lentiviral vectors were Gibson assembled between PmeI sites using primers: mCLEC14A-ECD; forward 5′-ACTAGCCTCGAGGTTTAAACATGAGGCCAGCGCTTGCC-3′ and reverse 5′-CACTCGATGAGGATCCGGAAGAGGTGTCGAAAGTCAGAGAAAC-3′, mouse Fc for fusion to mCLEC14A-ECD-forward 5′-CCTCTTCCGGATCCTCATCGAGTGTGCCCAGGGATTGTGGT-3′ and reverse 5′-CTGCAGCCCGTAGTTTTCATTTACCAGGAGAGTGGG-3′. mFc alone fused to CLEC14A SP; mCLEC14A SP-forward 5′-AGACTAGCCTCGAGGTTTAAACATGAGGCCAGCGCTTGC-3′ and mCLEC14A SP-reverse 5′-TGAGGATCCCTCCCCATTCCCTGGCCG-3′, mFc fused to SP; forward 5′-AATGGGGAGGGATCCTCATCGAGTGTG-3′ and reverse 5′-TCCTGCAGCCCGTAGTTTTCATTTACCAGGAGAGTGG-3′. mMMRN2^495^^–^^678^ inserted between engineered BamHI site separating the SP and mFc; forward 5′-CAGGGAATGGGGAGGGATCCCAAAGGGTCAACTCTGACG-3′ and reverse 5′-GGCACACTCGATGAGGATCCCAACTGTGGGTGCTGCTC-3′. Human and mouse MMRN2, human and mouse CLEC14A, CD248 and thrombomodulin amplified from IMAGE clones, mFc amplified from cDNA of C3 hybridomas. CD93 amplified from pCDNA3-CD93, a gift from Suzanne Bohlson. CLEC14A domain deletions are as previous.^4^

### Protein expression and purification

CLEC14A-ECD-Fc was expressed and purified as described.^3^ MMRN2^495^^–674^ and MMRN2^495^^–603^ with His tag or avitag were expressed in *E. coli* strain BL21-DE3 pLysS (Promega, Southampton, UK) by induction with 0.5 mM IPTG at OD_600_ 0.6 and grown at 18 °C overnight. The cell pellets were homogenized by high pressure lysis (17 000 p.s.i.) in Emulsiflex-C3 (4 °C; Avestin, Winchester, UK) in buffer; 50 mM Na_2_PO_4_ pH 7.4 400 mM NaCl, 10% (v/v) glycerol, 50 mM imidazole, 0.5 mM TCEP and complete EDTA-free protease inhibitors (Roche, Burgess Hill, UK), then loaded onto Nickel-NTA columns. Fractions eluted using 500 mM imidazole were purified by size exclusion chromatography on S200 columns on an AKTA FPLC machine (GE Healthcare) in buffer; 20 mM Tris pH8.0, 50 mM NaCl. Proteins were buffer exchanged into PBS and endotoxin removed using high capacity endotoxin removal columns (Thermo Fisher Scientific) then filter sterilized.

### Western and far western blotting

Standard protocols for western blotting were used. Far westerns involved incubating polyvinylidene fluoride membranes for 1 h with hCLEC14A-ECD-Fc (2 μg/ml), CD248-ECD-Fc (2 μg/ml), or MMRN2^FL^-transfected lysates (6 × 10^6^ HEK293T cells/ml of lysis buffer) (diluted 1:50). Epitope tags of each protein probe were detected with secondary antibodies as standard.

### Cell culture, transfections and transductions

HUVECs were isolated from umbilical cords collected at the Birmingham Women’s Hospital with consent and cultured as described.^2^ HUVEC experiments used at least three distinct preparations from different cords. HEK293T were cultured as described.^3^ Transfections and lentiviral transductions performed as previous.^4^

### Cell surface biotinylation and immunoprecipitation

Transfected HEK293T washed twice with PBS (Mg^2+^ and Ca^2+^), then EZ-Link Sulfo-NHS-Biotin (Thermo Fisher Scientific #21217) was incubated at 1 mg/ml in PBS for 30 min, biotinylation reaction quenched using 100 mM glycine and cells washed twice with PBS were immunoprecipitated with 2–5 μg of antibody or Fc-tagged protein as described.^3^

### Sequence alignment and structure prediction modelling

Protein sequences were aligned using a described algorithm.^37^ iTASSER servers were used to predict the three-dimensional molecular structure of CLEC14A-CTLD (Accession number Q86T13 residues 21–173). The model with the highest C-score (0.05) and organized structure was chosen.

### Immunofluorescence staining and analysis

HUVEC on gelatin-coated coverslips were cultured for 6 days, media replaced every 2 days, fixed and blocked as described without permeabilization.^38^ Incubated with anti-MMRN2 (Abnova, 4 μg/ml) and hFc, mCD248-ECD-Fc or CLEC14A-ECD-Fc (all 20 μg/ml) for 2 h. Detection antibodies; anti-human alexafluor-555 and anti-mouse alexafluor-488 incubated for 1.5 h. Human pancreatic tumour sections were cleared of paraffin, rehydrated and antigen retrieved in Tris-EDTA pH 9.0 for 1 h (96 °C), blocked in 2.5% horse serum PBS 30 min, anti-MMRN2 (Abcam, 0.45 μg/ml), anti-CLEC14A (R&D, 50 μg/ml), anti-CD248 B1.35 (1:5 dilution) 1 h. Detection antibodies: anti-rabbit alexafluor-647, anti-mouse alexafluor-488, anti-sheep alexafluor-546. Frozen tumour sections stained with anti-CD31 (MEC13.3, 75 ng/ml) and anti-rat alexafluor-488. Imaged using confocal microscope Zeiss LSM-780 with Argon and He/Ne lasers, 40x or 63x/1.40 water immersion objectives. Images displayed as maximum intensity projections. Vessel analysis was as described (Supplementary Figure S9).

### Flow cytometry

Detached HUVEC or transfected HEK293T (5 × 10^5^) were stained with antibody or recombinant protein (20 μg/ml). In blocking experiments, hCLEC14A-ECD-Fc was incubated with 2 × molar excess of antibody or recombinant protein for 1 h (4 °C). In GFP-tagged overexpressions, GFP+ cells were gated for analysis. Detection reagents; anti-hFc-FITC (1:100), streptavidin-PE (1:100), anti-mouse alexafluor-555 (1:100). The samples were analysed on a FACSCalibur (BD Biosciences).

### ELISA assay

The 96-well plates were coated with mCD248-ECD-Fc (400 ng) in PBS overnight (4 °C), blocked (PBS 3% (w/v) BSA), then MMRN2^FL^ transfected HEK293T lysates (6 × 10^6^ cells/ml) added diluted in PBS (1:50). CLEC14A-ECD-Fc (200 ng) or anti-His antibodies (200 ng), then for CLEC14A-ECD-Fc, C2 (400 ng) was incubated. Binding detected with anti-mouse-HRP (1:5000), visualized using BM Blue POD substrate (Roche).

### Adhesion assay

MMRN2^495−674^, MMRN2^495^^−^^603^ or BSA (2 μg) coated on 96-well plates overnight in PBS (37 °C). Then blocked (PBS 3% BSA) and dissociated HUVEC (50 000/well) added for 4 h (37 °C), washed five times, fixed (4% paraformaldehyde) and stained (0.5% crystal violet solution; Sigma-Aldrich). Images taken (Leica DM IL microscope and 2M Xli camera) and absorbance measured at 590 nm.

### Matrigel and co-culture assays

Performed as described,^3, 38, 39^ recombinant proteins added at 20 μg/ml in PBS.

### Mouse tumour implantation assays

A total 10^6^ transduced LLC were subcutaneously injected into the right flank of male C57BL/6 mice aged 8−10 weeks old. After 2 weeks or when tumour size limit of 1200 mm^3^ was reached, the animals were killed, tumours excised and weights determined. The mice were housed at the Birmingham Biomedical Services Unit. Animal experimentation was carried out in accordance with Home Office License PPL70/8704 held by RB.

### Statistical analysis

Statistical tests were calculated using Graphpad Prism software and were all two-tailed tests, all replicates are biological replicates and *n* numbers are stated in the figure legends.
