# Supplementary material for: Multimerin-2 is a ligand for group 14 family C-type lectins CLEC14A, CD93 and CD248 spanning the endothelial pericyte interface
Source: Oncogene. 2017 Jul 3;36(44):6097–108. doi: 10.1038/onc.2017.214 (PMC5671938; doi:10.1038/onc.2017.214)
Supplement: Supplementary Information [file onc2017214x1.pdf]

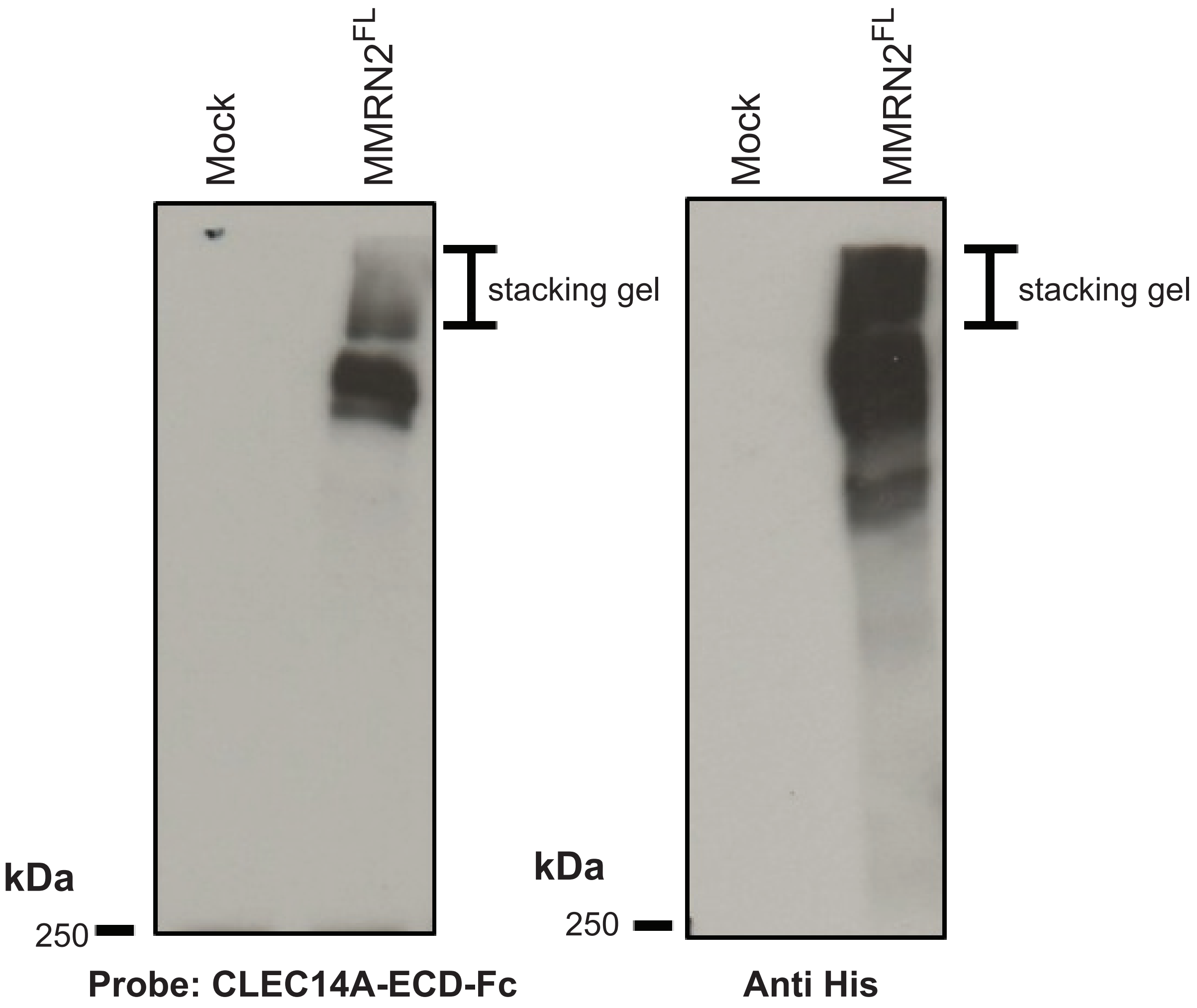

Figure S1. CLEC14A-ECD binds MMRN2 under non-reduced conditions. HEK293T transfected with MMRN2^{FL} were lysed and separated on a 6% polyacrylamide gel under non-reducing conditions. MMRN2^{FL} forms large molecular weight complexes that barely enter the stack gel. CLEC14A far westerns show binding to MMRN2^{FL} multimers. His tag blot is included to show expression of MMRN2^{FL}.

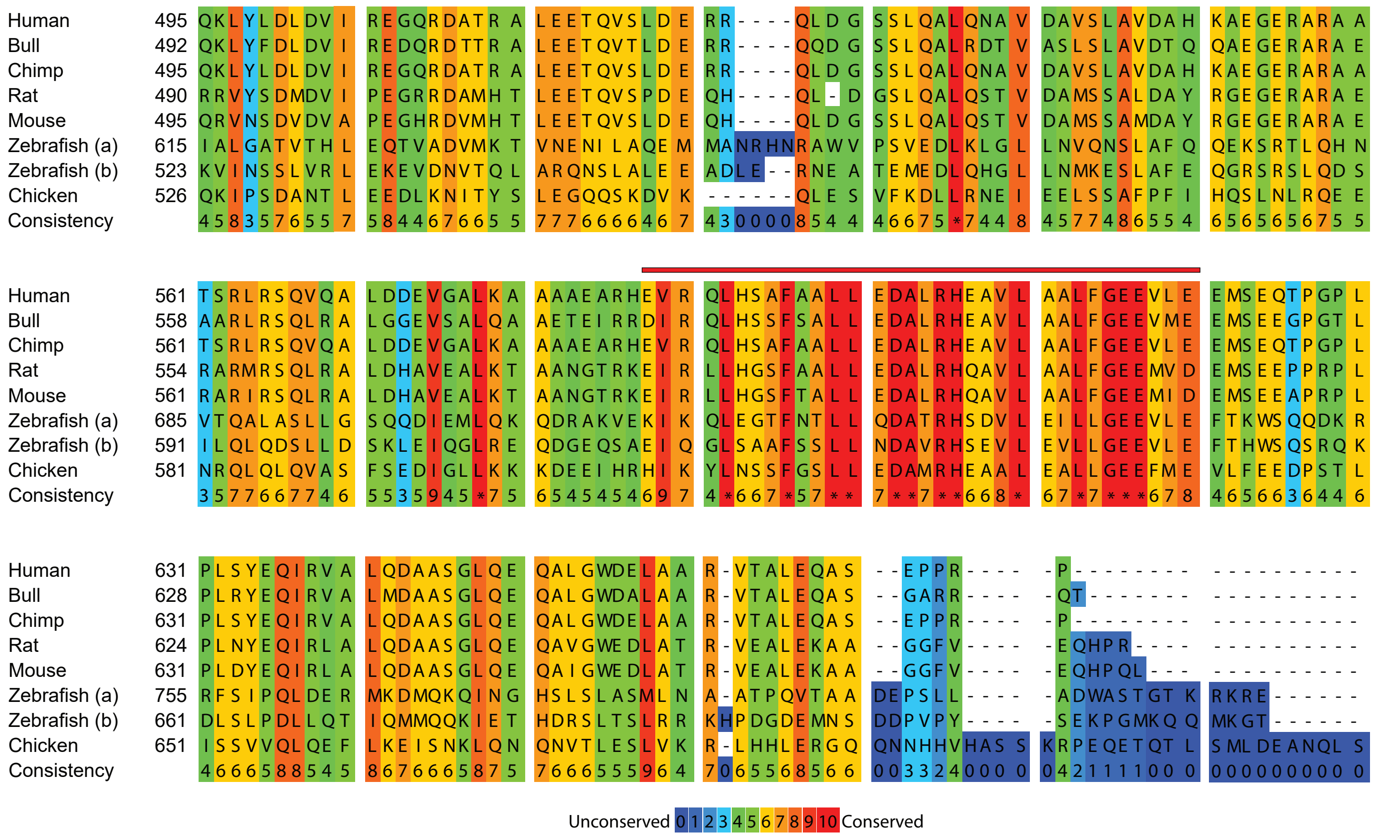

Figure S2. Amino acid sequence alignments of human MMRN2$^{495\text{-}674}$ and corresponding regions from different species. PRALINE (1) generated amino acid alignments of MMRN2 regions from human (Accession no. Q9H8L6), bull (E1BJW3), chimp (H2Q273), rat (D4ABX6), mouse (A6H6E2), chicken (E1BTB7) and two zebrafish proteins MMRN2a (zebrafish (a)) (F1R6V5) and MMRN2b (zebrafish (b)) (E7FG77). This reveals a highly conserved region between residues 588-620 (red line) of human MMRN2. This region contains 72% amino acid identity between human and mouse MMRN2. Residues are labelled for conservation by colour. Blue = unconserved, red = conserved.

1. Simossis VA, Heringa J. PRALINE: a multiple sequence alignment toolbox that integrates homology-extended and secondary structure information. *Nucleic Acids Res* 2005; **33:** W289-W294.

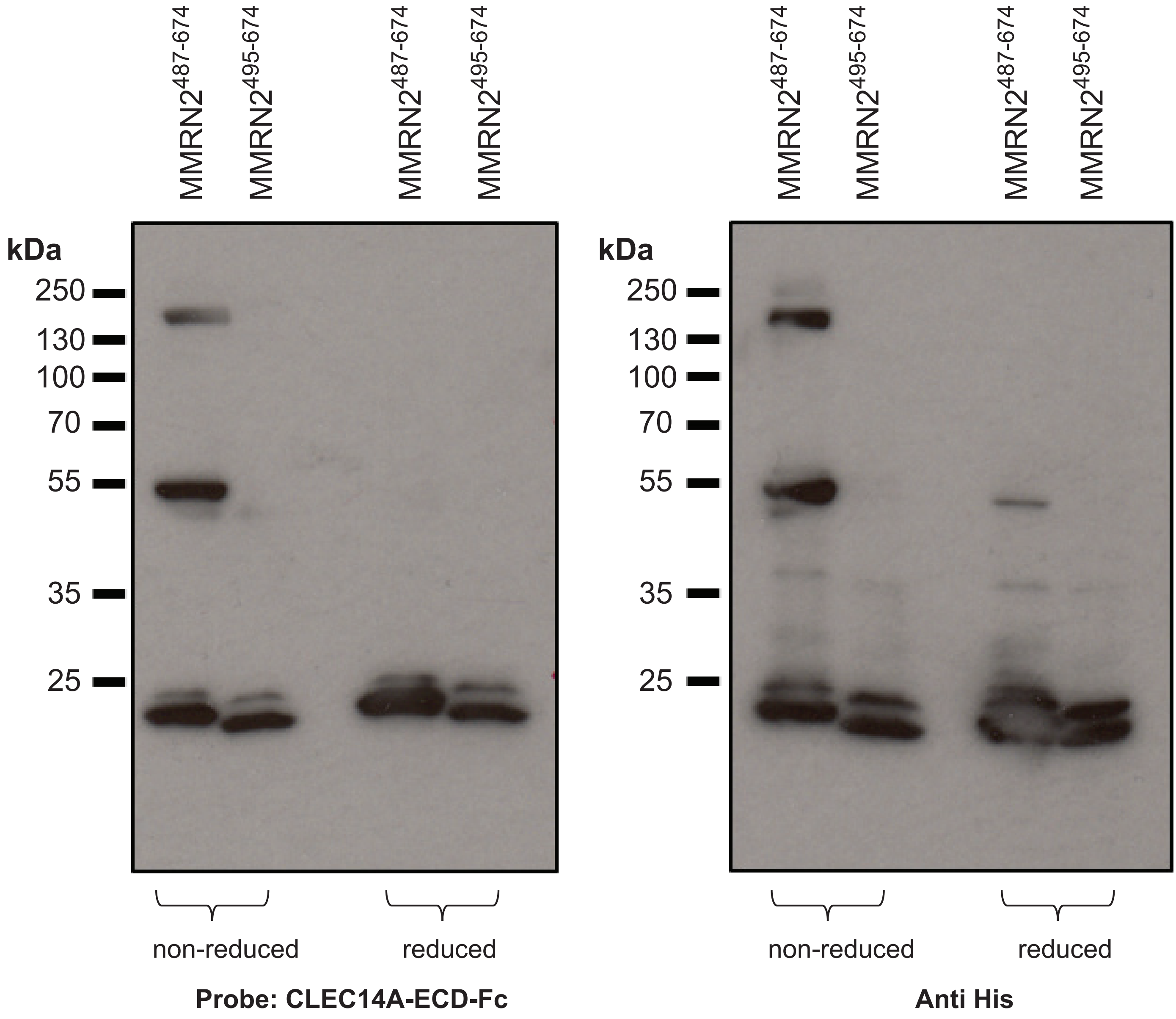

Figure S3. CLEC14A-ECD-Fc binds MMRN2$^{487\text{-}674}$ and MMRN2$^{495\text{-}674}$. Each MMRN2 fragment was transfected into HEK293T, lysed and subjected to far western blotting using CLEC14A-ECD-Fc, this revealed binding to both fragments under reduced (R) and non-reduced (NR) conditions. Under NR conditions MMRN2$^{487\text{-}674}$ forms large molecular weight multimers that also bind CLEC14A. His tag blot included to show expression of each protein.

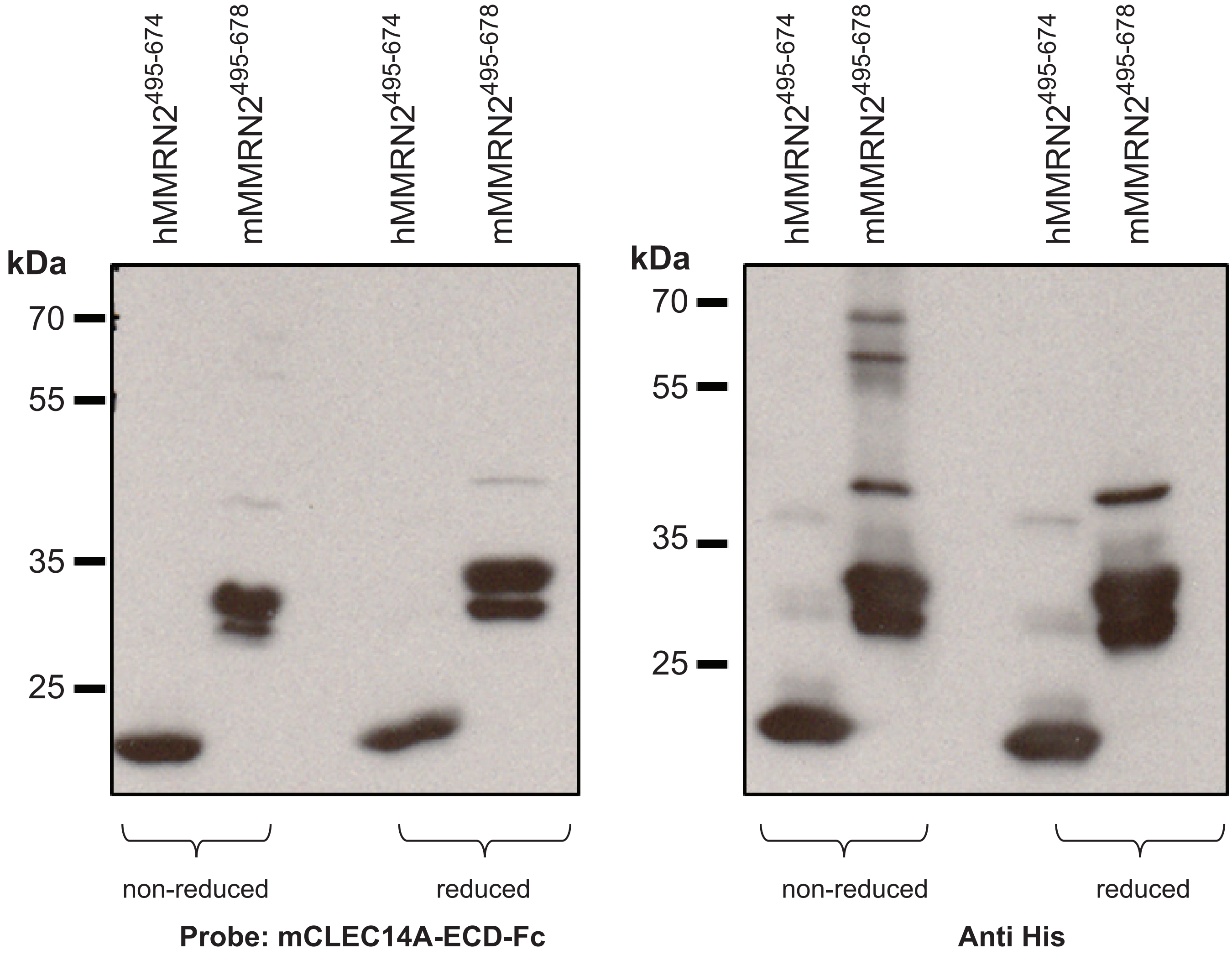

Figure S4. Mouse CLEC14A directly binds to human MMRN2$^{495\text{-}674}$ and mouse MMRN2$^{495\text{-}678}$. Far western blots using mouse CLEC14A-ECD-Fc shows specific binding to human MMRN2$^{495\text{-}674}$ and equivalent mouse MMRN2$^{495\text{-}678}$, under reduced and non-reduced conditions. His tag blot is included to show expression of each protein fragment. Mouse MMRN2$^{495\text{-}678}$ likely appears at a higher molecular weight due to a potential glycosylation site within this region.

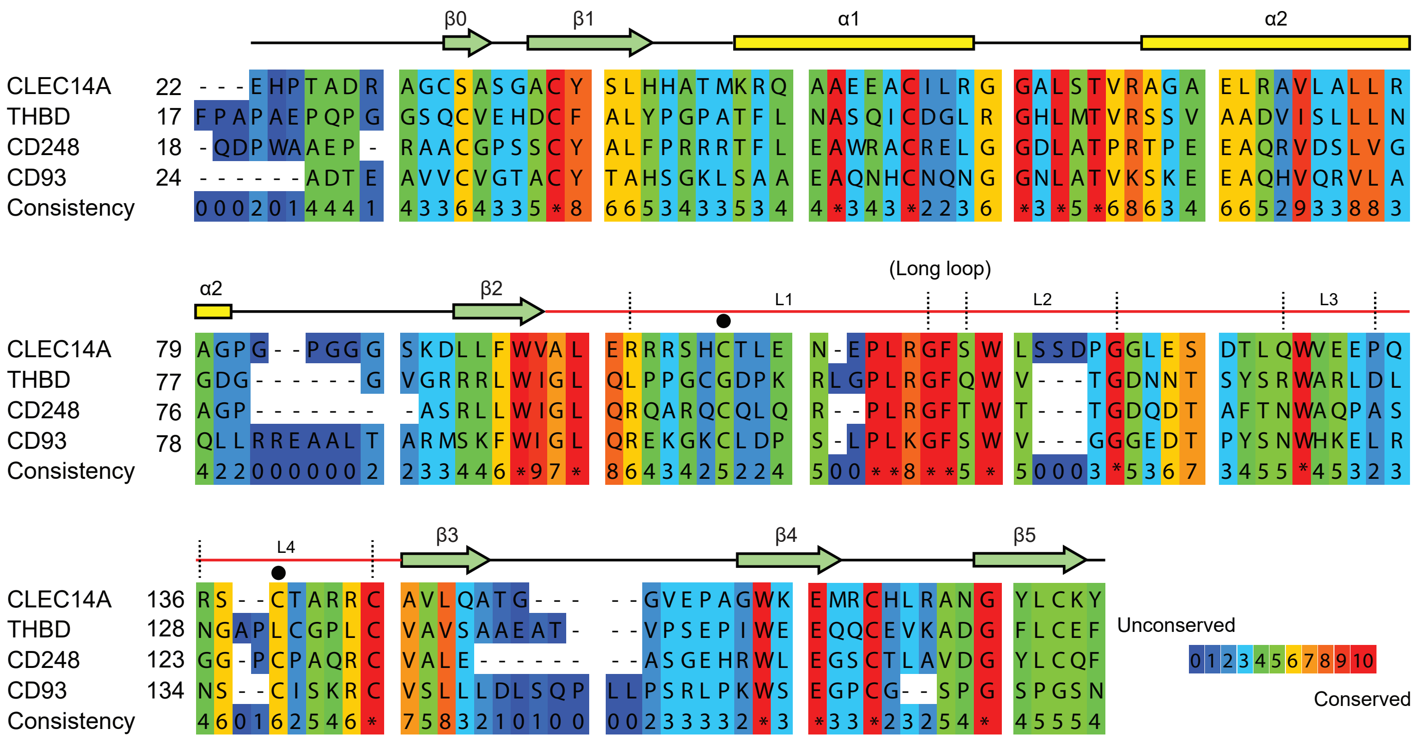

Figure S5. Amino acid sequence alignments of CTLD group 14 family members. Human CLEC14A (Accession no. Q86T13), CD93 (Q9NPY3), THBD (P07204) and CD248 (Q9HCU0) CTLD sequences were aligned using PRALINE without inclusion of signal peptides. The α-helices (yellow rectangle) and β-sheets (green arrow) are mapped onto the alignment corresponding to their position within the predicted CLEC14A-CTLD model. There are also four predicted loops L1-L4 mapped within the long-loop region (red) as described for tetranectin CTLD. The L1 loop contains the 97-108 region and the non-canonical cysteine 103. The L4 contains the other non-canonical cysteine C138. Each cysteine residue within the long-loop region is labelled with a black dot.

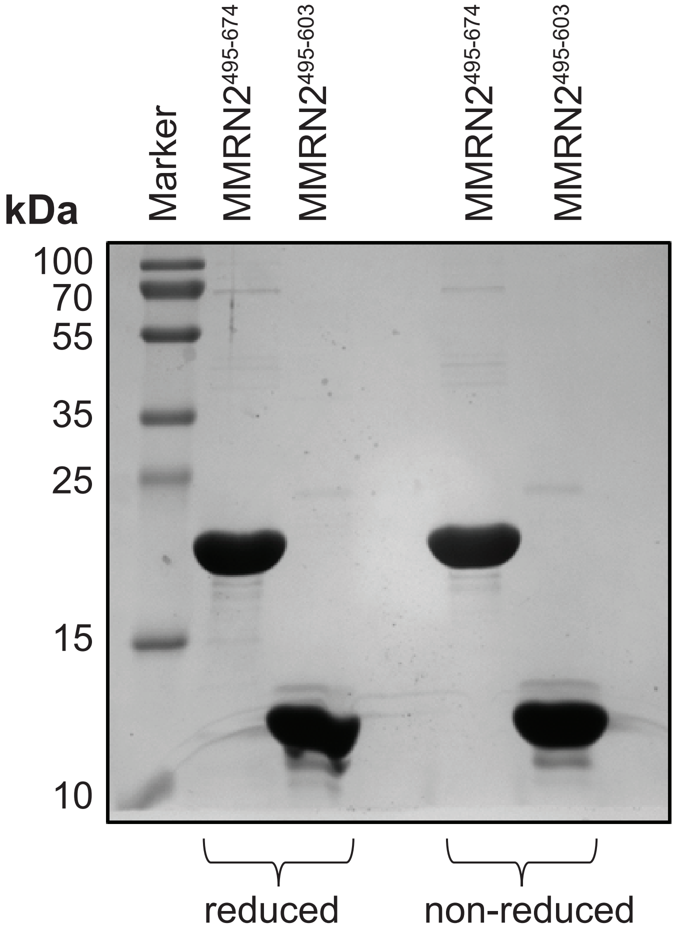

Figure S6. Coomassie stain of *E.coli* expressed and purified MMRN2$^{495\text{-}674}$ and MMRN2$^{495\text{-}603}$. SDS-PAGE and coomassie staining reveals both recombinant protein preparations are of high purity. MMRN2$^{495\text{-}674}$ resolves at ~20 kDa and MMRN2$^{495\text{-}603}$ at ~12.5 kDa.

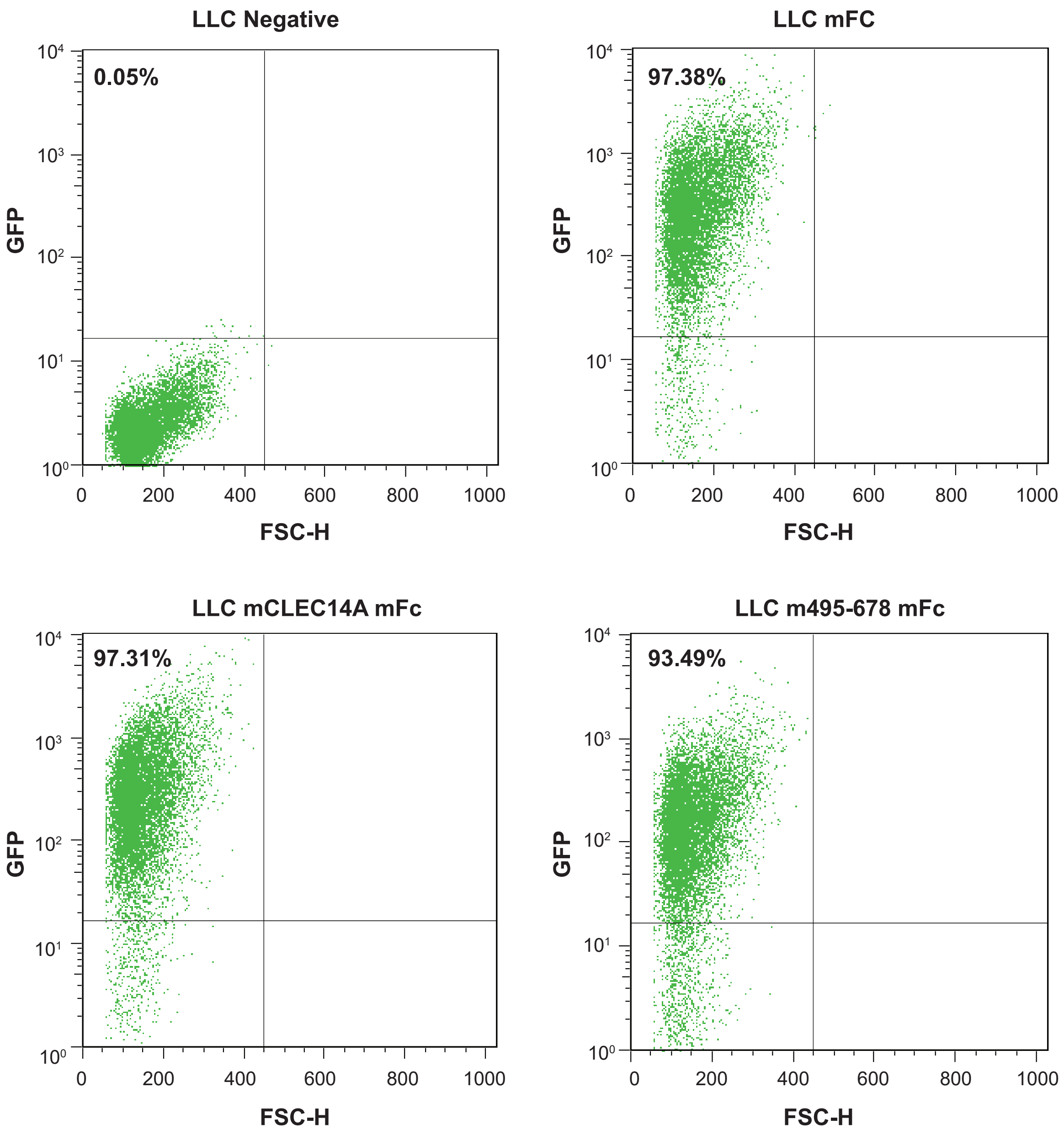

Figure S7. Flow cytometry analysis of Lewis lung carcinoma transductants. Levels of green fluorescent protein (GFP) were compared to non-transduced cells (LLC –ve). All transduction efficiencies were over 90%.

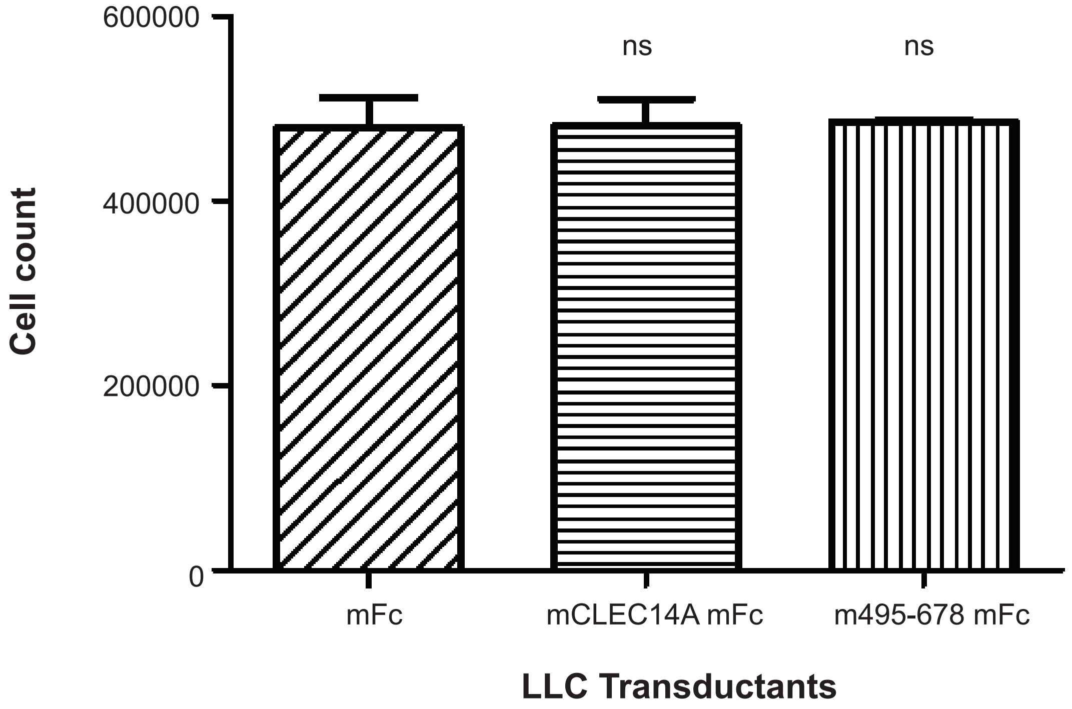

Figure S8. Cell proliferation is unaffected between Lewis lung carcinoma transductants *in vitro*. Cells were plated and allowed to proliferate for 2 days and then counted using a coulter counter. This experiment was performed three times. There was no large difference in the cell count between each LLC transfectant. Mann Whitney test, mFc vs. mC14A mFc $p = 1.0$, mFc vs. m495-678 $p = 0.7$ $n=3$. Error bars represent SEM.

A

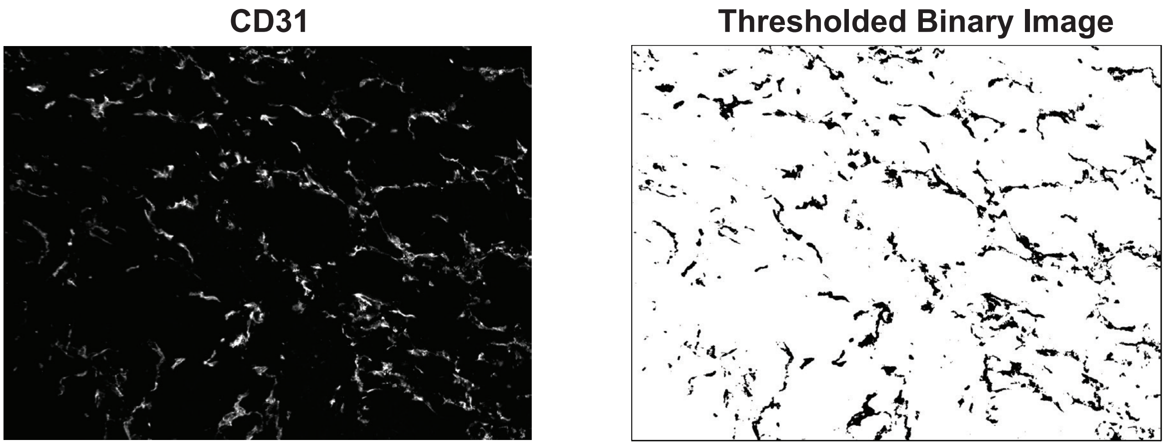

B

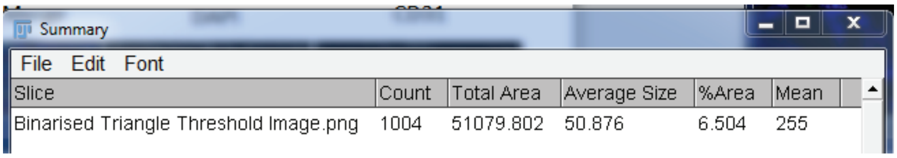

Slice	Count	Total Area	Average Size	%Area	Mean
Binarised Triangle Threshold Image.png	1004	51079.802	50.876	6.504	255

C

```
Macro:
//run("Threshold...");
setAutoThreshold("Triangle dark");
setOption("BlackBackground", false);
run("Convert to Mask");
run("Analyze Particles...", "display summarize");
```

Figure S9. CD31+ vessel analysis. (A) For each image CD31 staining thresholds were set using an automated Triangle thresholding method using ImageJ Fiji. (1). (B) The subsequent binary image was then analysed using the ‘Particle Analysis’ plugin to measure area, average ‘vessel’ size, and percentage area. (C) The ImageJ macro that was used is displayed.

1. Zack GW, Rogers WE, Latt SA. Automatic measurement of sister chromatid exchange frequency. *Journal of Histochemistry and Cytochemistry* 1977; **25:** 741–753.

**MMRN2 fragment**	**CLEC14A binding**	**CD93 binding**	**CD248 binding**
MMRN2^{FL}	+	+	+
MMRN2^{EMI-CC}	+	Not tested	+
MMRN2^{CC-C1q}	+	Not tested	+
MMRN2^{CC}	+	Not tested	+
MMRN2$^{133-486}$	-	Not tested	+
MMRN2$^{487-820}$	+	Not tested	-
MMRN2$^{487-674}$	+	Not tested	-
MMRN2$^{495-674}$	+	+	-
MMRN2$^{495-603}$	-	-	-
MMRN2$^{530-624}$	+	Not tested	-
MMRN2$^{604-674}$	-	Not tested	-

Supplementary Table 1. A summary of which CTLD group 14 family members bind to which regions of MMRN2.
